# A Novel Viral Assembly Inhibitor Blocks SARS-CoV-2 Replication in Airway Epithelial Cells

**DOI:** 10.21203/rs.3.rs-2887435/v1

**Published:** 2023-05-17

**Authors:** Satish Pillai, Li Du, Fred Deiter, Mohamed Bouzidi, Jean-Noel Billaud, Simmons Graham, Dabral Prerna, Suganya Selvarajah, Anuradha Lingappa, Maya Michon, Shao Yu, Kumar Paulvannan, Vishwanath Lingappa, Homer Boushey, John Greenland

**Affiliations:** Vitalant Research Institute / UCSF; Vitalant Research Institute/UCSF; Veterans Administration Health Care System/UCSF; Vitalant Research Institute; QIAGEN Digital Insights; Vitalant Research Institute / UCSF; Vitalant Research Institute / UCSF; Prosetta Biosciences Inc; Prosetta Biosciences Inc; Prosetta Biosciences Inc; Prosetta Biosciences Inc; Prosetta Biosciences Inc; Prosetta Biosciences Inc; University of California San Francisco; University of California, San Francisco

## Abstract

The ongoing evolution of SARS-CoV-2 to evade vaccines and therapeutics underlines the need for novel therapies with high genetic barriers to resistance. The small molecule PAV-104, identified through a cell-free protein synthesis and assembly screen, was recently shown to target host protein assembly machinery in a manner specific to viral assembly. Here, we investigated the capacity of PAV-104 to inhibit SARS-CoV-2 replication in human airway epithelial cells (AECs). Our data demonstrate that PAV-104 inhibited > 99% of infection with diverse SARS-CoV-2 variants in primary and immortalized human AECs. PAV-104 suppressed SARS-CoV-2 production without affecting viral entry or protein synthesis. PAV-104 interacted with SARS-CoV-2 nucleocapsid (N) and interfered with its oligomerization, blocking particle assembly. Transcriptomic analysis revealed that PAV-104 reversed SARS-CoV-2 induction of the Type-I interferon response and the ‘maturation of nucleoprotein’ signaling pathway known to support coronavirus replication. Our findings suggest that PAV-104 is a promising therapeutic candidate for COVID-19.

## Introduction

Severe acute respiratory syndrome coronavirus-2 (SARS-CoV-2), the etiological agent of the ongoing COVID-19 pandemic, belongs to a highly contagious betacoronavirus^1^. The high transmission and variation of SARS-CoV-2 poses an ongoing threat to global public health. Despite multiple vaccine options, only 65.4% of people are currently fully vaccinated worldwide, due in part to lack of vaccine access as well as behavioral resistance to vaccination. Moreover, many people remain at high risk for severe COVID-19 due to decreased vaccine efficacy and increased risk of respiratory failure associated with immune compromise.

For the treatment of SARS-CoV-2, anti-SARS-CoV-2 monoclonal antibodies (mAbs) that target the spike protein represent one class of therapeutic candidates approved by the FDA for COVID-19 patients^2,3^. However, the effi cacy of anti-SARS-CoV-2 mAbs is negligible in the face of currently circulating viral variants^4^. Beyond mAbs, antiviral small-molecule drugs have been developed that target specific parts of the viral life cycle to prevent infectivity, severe illness and death attributed to COVID-19. Three antiviral agents are currently authorized by the FDA for the treatment of COVID-19: viral RNA-dependent RNA polymerase (RdRp) inhibitors, remdesivir and molnupiravir^4–6^ and a viral 3C-like protease inhibitor, paxlovid, which consists of nirmatrelvir and ritonavir^7^. Clinical studies have shown that remdesivir is not associated with statistically significant clinical benefits^8^. *In vitro* studies have shown that molnupiravir is used as a substrate by host RNA polymerases including the mitochondrial DNA-dependent RNA polymerase^9^. Paxlovid treatment is often associated with COVID-19 rebound following the treatment cycle^10^. Taken together, these realities illustrate the need for effective and broad-spectrum antiviral drugs for COVID-19 with minimal off-target effects.

Recent studies have focused on the SARS-CoV-2 viral life cycle to find additional targets for drug therapy, providing candidate SARS-CoV-2 entry and attachment inhibitors^11,12^, novel viral protease inhibitors^13,14^, and N assembly inhibitors that reduce viral nucleocapsid (N)-genome RNA interaction^15,16^. However, data are limited describing agents that selectively inhibit the late stage of the SARS-CoV-2 viral life cycle of particle formation/budding^17^. The viral assembly/budding process is a dynamic program dependent on transient multi-protein assembly complexes^18^. One approach to identifying these host-viral drug targets is by interrogation of the pathway of viral assembly/budding in cell-free protein synthesis and assembly systems^19–21^. Small molecules have been identified in this manner that are active against various viral families including those of rabies virus, HIV-1, and influenza virus^22–24^.

The small molecule PAV-104, identified through a moderate-throughput screen involving cell-free protein synthesis and assembly (CFPSA), was recently shown to target host protein assembly machinery in a manner specific to viral assembly^23^. This compound has minimal host toxicity, and once-daily oral dosing in rats that achieves > 200-fold of the 90% effective concentration (EC_90_) in blood. Pharmacokinetic studies of PAV-104 in rats showed a lung to plasma ratio of 0.3 after oral administration^23^. The chemotype shows broad activity against respiratory viral pathogens, including *Orthomyxoviridae*, *Paramyxoviridae*, *Adenoviridae*, *Herpesviridae*, and *Picornaviridae*, with low susceptibility to evolutionary escape.

Here, we evaluated the antiviral effect of PAV-104 against SARS-CoV-2 infection in immortalized and primary human airway epithelial cells (AECs), and elucidated the mechanism underlying this antiviral activity. Our results show that PAV-104 treatment potently inhibits the replication of diverse SARS-CoV-2 variants. We further demonstrate that PAV-104 specifically inhibits the late stages of the SARS-CoV-2 replication cycle, perturbing the oligomerization of viral N, thereby blocking viral capsid assembly and budding. PAV-104 treatment also reverses SARS-CoV-2 induction of the Type-I interferon response and the ‘maturation of nucleoprotein’ signaling pathway which supports coronavirus replication within the target cell. Together, our data suggest that PAV-104 is a promising therapeutic candidate for COVID-19, and its mechanism of action is distinct from existing clinical strategies.

## Results

### PAV-104 suppresses SARS-CoV-2 infection in Calu-3 cells

**Figure 1:**
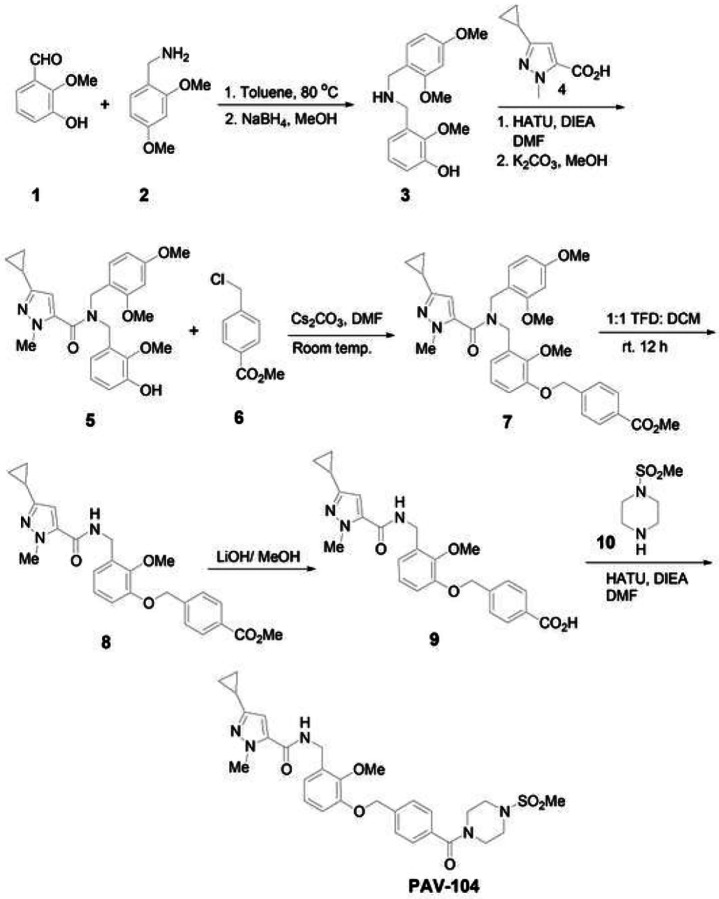
Synthesis and molecular structure of PAV-104. A detailed description of PAV-104 synthesis and preparation is provided in the Methods section.

**Figure 2:**
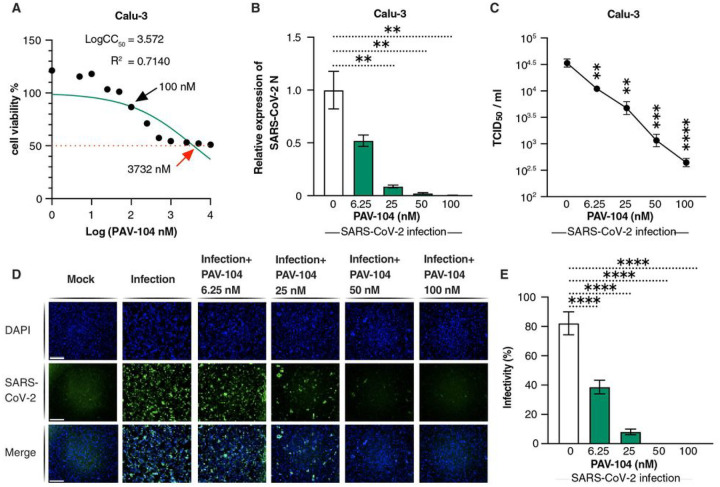
PAV-104 decreases virus production in SARS-CoV-2-infected Calu-3 cells. (A) MTT assay was performed on Calu-3 cells to examine the cellular toxicity of PAV-104. Relative cell viability was displayed based on the PAV-104-untreated control (set at 100%). The red arrow represents the CC_50_ value (3732 nM) of PAV-104. (B) Anti-SARS-CoV-2 activity of PAV-104 in Calu-3 cells was measured by RT-qPCR targeting the *N* genes. Cells were pretreated with PAV-104 at the indicated concentrations for 1 hour, followed by infection with SARS-CoV-2 (MOI=0.01) for 24 h in the presence of PAV-104. RNA isolation and RT-qPCR assay was performed 24 h post-infection. (C) The SARS-CoV-2 titer (TCID_50_) was measured after treatment with varying doses of PAV-104 as described in (B). (D) Immunofluorescence staining of Calu-3 cells with DAPI (blue) was performed at 72 h postinfection. Cells were pretreated with PAV-104 at the indicated concentrations, followed by infection with SARS-CoV-2-GFP virus. Scale bar, 500 μm. (E) Quantification of SARS-CoV-2 infected cells (GFP positive cells) in Calu-3 cells (shown in panel D). Data are representative of the results of three independent experiments (mean ± SEM). Statistical significance was analyzed by *t* test. *p*≤*0.05* [*], *p* ≤ *0.01* [**], *p*≤*0.001* [***], *p*≤*0.0001* [****].

The synthesis and molecular structure of PAV-104 (Fig. 1), a small molecule drug recently associated with pan-respiratory virus antiviral activity^23^, is described in detail in the Methods section. To investigate the impact of PAV-104 on SARS-CoV-2 infection, we first determined the cytotoxicity of PAV-104, to optimize dosing in Calu-3 cells. The 50% cytotoxic concentration (CC_50_) value detected by MTT assay was 3732 nM for PAV-104 in Calu-3 cells (Fig. 2A). Next, we evaluated the effects of PAV-104 on SARS-CoV-2 replication at 6.25 nM, 25 nM, 50 nM, and 100 nM concentrations. Calu-3 cells were pretreated for one hour with PAV-104 followed by infection with SARS-CoV-2 at an MOI of 0.01, and PAV-104 was maintained in the media until 24 hours following viral infection. SARS-CoV-2 replication, as measured by quantitation of viral nucleocapsid (*N*) gene expression, was decreased significantly by treatment with PAV-104 in a dose dependent manner (*p* < *0.01*) (Fig. 2B). Similarly, release of infectious virus in the supernatant was suppressed significantly by PAV-104 in a dose dependent manner, as measured by median tissue culture infectious dose (TCID_50_) (*p* < *0.01*) (Fig. 2C), with up to 75-fold reduction at the highest concentration of PAV-104. The inhibition of virus production by PAV-104 was confirmed by specific staining of viral N using an immunofluorescence assay (IFA) (Fig. 2D, E). Taken together, these data demonstrate that PAV-104 decreases SARS-CoV-2 viral production in susceptible Calu-3 cells.

### PAV-104 and remdesivir potently inhibit SARS-CoV-2 replication

**Figure 3:**
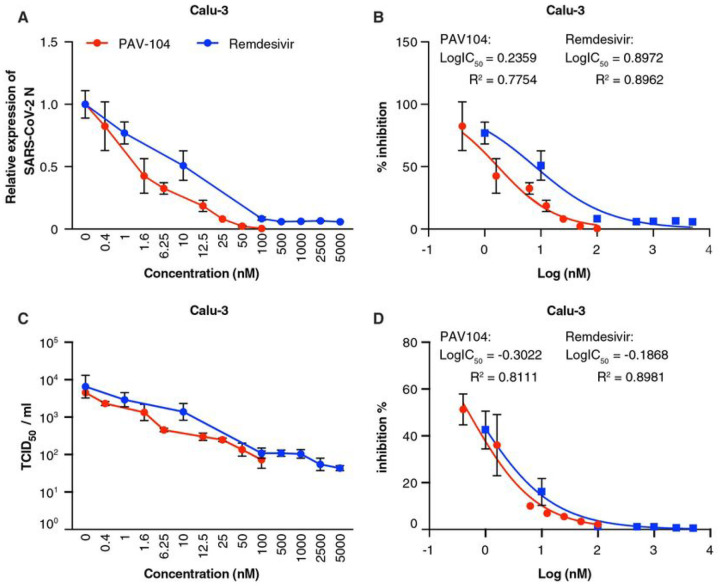
PAV-104 inhibits SARS-CoV-2 replication in Calu-3 cells more potently than remdesivir. (A) Reduction of SARS-CoV-2 replication by PAV-104 and remdesivir in Calu-3 cells, as determined by RT-qPCR targeting the *N* gene. Calu-3 cells were pretreated with DMSO, PAV-104, or remdesivir for one hour, then infected with SARS-CoV-2 at an MOI of 0.001. Supernatants and cells were collected at 48 hpi. (B) Percent inhibition of SARS-Cov-2 replication by PAV-104 and remdesivir in Calu-3 cells, as determined by RT-qPCR (PAV-104: EC_50_ = 1.7 nM, EC_90_ = 23.5 nM; remdesivir: EC_50_ = 7.9 nM, EC_90_ = 218.1 nM). (C) Reduction of SARS-CoV-2 replication by PAV-104 and remdesivir in Calu-3 cells, as determined by infectious viral titer. (D) Percent inhibition of SARS-CoV-2 replication by PAV-104 and remdesivir in Calu-3 cells, as determined by infectious viral titer (PAV-104: EC_50_ = 0.5 nM, EC_90_ = 10.3 nM; remdesivir: EC_50_ = 0.65 nM, EC_90_ = 19.2 nM). Data are representative of the results of three independent experiments (mean ± SEM).

Remdesivir is the first approved small-molecule anti-SARS-CoV-2 drug for COVID-19 treatment and shows potent anti-SARS-CoV-2 activity in Calu-3 cells^25,26^. To compare the anti-SARS-CoV-2 effi cacies of PAV-104 with remdesivir in Calu-3 cells, Calu-3 cells were infected with SARS-CoV-2 for 48 hours at the MOI of 0.001 and treated with varying doses of PAV-104 or remdesivir. Cells and supernatants were harvested for quantification of *N* expression by RT-qPCR and quantification of infectious viral titers by TCID_50_. Both compounds displayed dose-dependent inhibition of viral replication (Fig. 3A, B, C, D). Remdesivir inhibited SARS-CoV-2 with an EC_50_ value of 7.9 nM and a 90% maximal effective concentration (EC_90_) value of 218.1 nM (Fig. 3B). PAV-104 was more potent (EC_50_ = 1.7 nM and EC_90_ = 23.5 nM) than remdesivir, as determined by RT-qPCR (*p* < *0.05*) (Fig. 3B). EC_50_ value determined by quantification of infectious virus in the supernatants showed a similar trend (Fig. 3D). Thus, PAV-104 inhibits SARS-CoV-2 more potently than remdesivir in Calu-3 cells.

### PAV-104 is a highly potent antiviral inhibitor of SARS-CoV-2 in primary airway epithelial cells

**Figure 4:**
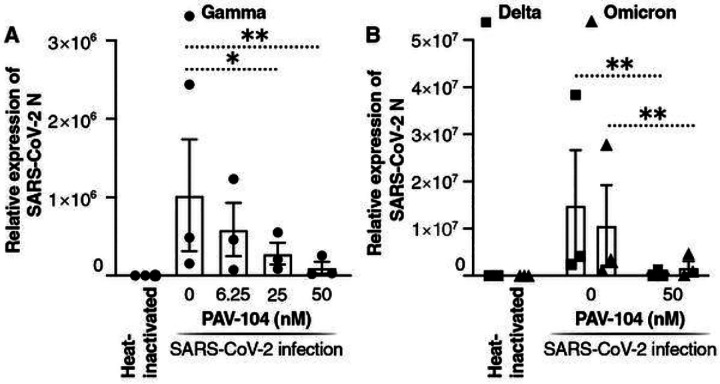
PAV-104 inhibits the replication of SARS-CoV-2 variants in human primary airway epithelial cells. (A) Antiviral activity of PAV-104 against SARS-CoV-2 in primary AECs, as determined by RT-qPCR. ALI-cultured primary AECs were pre-incubated with DMSO or PAV-104 at indicated concentrations for one hour and were then infected with heat-inactivated virus and SARS-CoV-2 (lineage P.1, MOI=0.1) at the apical and basal compartment for two hours. Cells were then washed and supplemented with fresh media containing DMSO or PAV-104. Cells were collected for RNA isolation and RT-qPCR at 36 hpi. Each color represents data from one donor. (B) Antiviral activity of PAV-104 against SARS-CoV-2 variants (Delta and Omicron) in primary AECs, as determined by RT-qPCR. Each color represents data from one donor. Data are representative of the results of three independent experiments (mean ± SEM). Statistical significance was analyzed by paired *t* tests. *p*≤*0.05* [*], *p* ≤ *0.01* [**], *p*≤*0.001*[***], *p*≤*0.0001* [****].

Upper and lower airways in humans are known to be the first gateway for SARS-CoV-2 infection^27^. To investigate the antiviral activity of PAV-104 against SARS-CoV-2 in human primary airway epithelial cells (AECs), we performed antiviral assays in air/liquid interface (ALI)-cultured AECs, which is useful in modeling the *in vivo* effects of PAV-104 on SARS-CoV-2 infection *ex vivo*^28^. We pretreated primary AECs from three healthy donors with PAV-104 and then infected them with the SARS-CoV-2 Gamma variant (Pango lineage designation P.1) for 36 hours. In PAV-104-treated, SARS-CoV-2-infected AECs cultures, there was > 99% inhibition of infection with PAV-104 treatment at the highest tested concentration (*p* < *0.01*) (Fig. 4A). We also tested the antiviral effect of PAV-104 on the emerging SARS-CoV-2 variants, Delta and Omicron, in AECs. Administration of PAV-104 also significantly reduced Delta and Omicron replication in primary AECs (*p* < *0.01*) (Fig. 4B). Together, these data demonstrate that PAV-104 exerts potent antiviral activity against a broad range of circulating SARS-CoV-2 variants in primary AECs.

### PAV-104 interferes with post-entry steps of the SARS-CoV-2 life cycle

**Figure 5:**
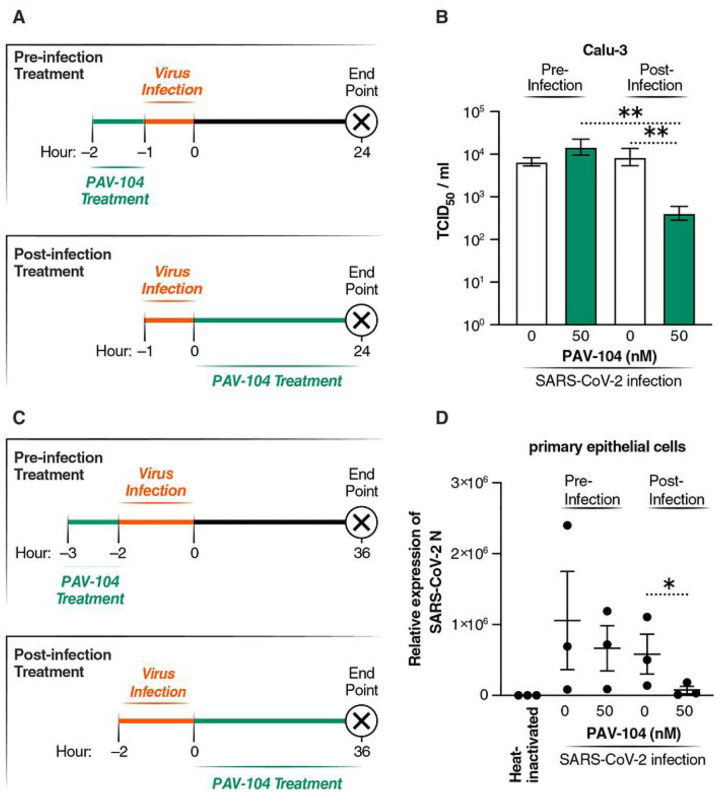
PAV-104 inhibits SARS-CoV-2 replication at a post-entry step of the viral life cycle. (A) Schematic timeline of PAV-104 treatment in Calu-3 cells. Calu-3 cells were incubated with PAV-104 or infected with SARS-CoV-2 at indicated time points as the diagram shows. (B) Virus production (measured as viral titer) in Calu-3 cells treated with PAV-104 at indicated doses and time points. (C) Schematic timeline of PAV-104 treatment in ALI-cultured primary AECs. Primary AECs were treated with PAV-104 or infected with SARS-CoV-2 at indicated time points. (D) Virus production (measured as viral *N* gene expression by RT-qPCR) in primary AECs treated with PAV-104 at indicated doses and time points. Heat-inactivated SARS-Cov-2 treatment was used for normalization. Data are representative of the results of three independent experiments (mean ± SEM). Statistical significance was analyzed by *t* test or paired *t* test. *p*≤*0.05* [*], *p*≤*0.01* [**], *p*≤*0.001*[***], *p*≤*0.0001* [****].

Since PAV-104 was identified based on the inhibition of the viral particle assembly/budding process, we next sought to determine whether PAV-104 inhibits SARS-CoV-2 replication by acting on a post-entry step of the SARS-CoV-2 viral replication cycle as expected. We treated Calu-3 cells with PAV-104 at the concentration of 50 nM before or after virus infection. Protocols are illustrated in Fig. 5A. Pre-infection treatment with PAV-104 did not inhibit infectious virus release into culture supernatants (Fig. 5B), indicating that PAV-104 does not act on early steps in the SARS-CoV-2 life cycle (e.g. viral attachment and entry). Post-infection treatment with PAV-104 did strikingly reduce SARS-CoV-2 viral titer in the supernatant by measuring TCID_50_ (*p* < *0.01*), as compared to post-infection treatment with DMSO (negative control) or pre-infection treatment with PAV-104 (Fig. 5B). Consistent with these data, SARS-CoV-2 replication in primary AECs was decreased significantly by treatment with PAV-104 post viral infection (*p* < *0.05*) (Fig. 5C, D). These results suggest that PAV-104 activity can be entirely attributed to blocking the late stage of the SARS-CoV-2 viral life cycle after viral entry.

### PAV-104 blocks SARS-CoV-2 viral particle formation

**Figure 6:**
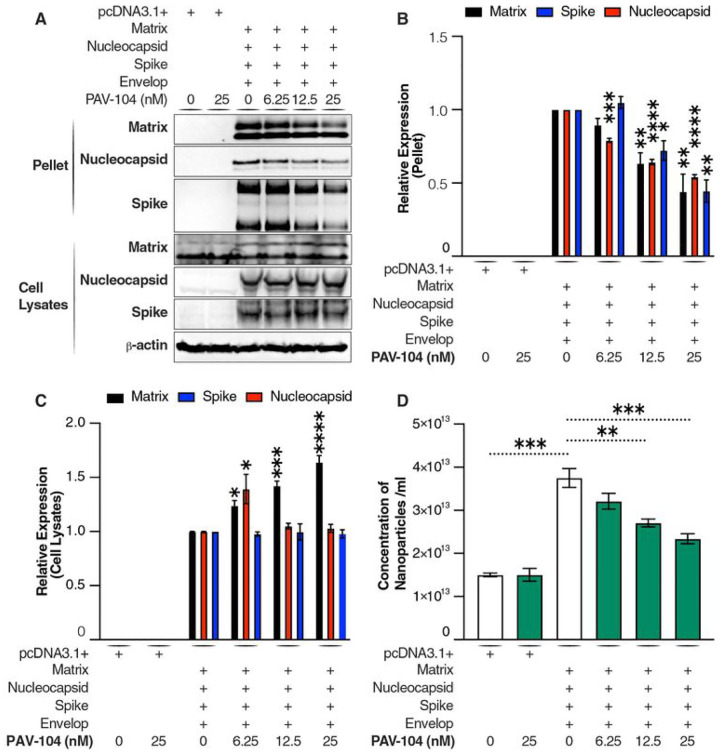
PAV-104 blocks SARS-CoV-2 viral assembly/budding. (A) Western blot analysis of structural protein expression in cell lysates and ultracentrifuged pellets. HEK293T cells were transfected with plasmids encoding the proteins indicated at the top. Western blots were performed with the primary antibodies indicated on the left of the blots. Anti-β-actin antibody was used as a loading control. (B and C) Relative quantification of the indicated protein from western blot (A). β-actin was used as a loading control for cell lysates and pellets. (D) Quantification of SARS-CoV-2 VLPs by nanoparticle tracking analysis. HEK293T cells were transfected with plasmids encoding the proteins indicated at the top. VLPs containing nanoparticles in the ultracentrifuged pellets from cell culture supernatants were diluted to a concentration in the range of 10^7^–10^9^/ml and examined using a NanoSight NS300 (NanoSight, Ltd) equipped with a 405 nm laser. Data are representative of the results of three independent experiments (mean ± SEM). Statistical significance was analyzed by *t* test or paired *t* test. *p*≤*0.05* [*], *p*≤ *0.01* [**], *p*≤*0.001* [***], *p*≤*0.0001* [****]

Transient coexpression of four SARS-CoV-2 structural proteins (N, M, E, and S) in cell culture has been shown to produce assembling virus-like particles (VLPs), which can be used to study the viral life cycle such as assembly/budding, egress, and entry^29,30^. To explore whether PAV-104 results in the inhibition of SARS-CoV-2 viral formation/budding, we quantified production of SARS-CoV-2 structural proteins in VLPs from cell culture supernatants of transfected HEK-293T cells treated with PAV-104 or DMSO. Viral assembly was quantified by western blot and nanoparticle tracking analysis (NTA) of extracellular vesicles and viral particles. Western blots were performed on proteins from the pellet after ultracentrifugation of transfected cell lysates and culture supernatants. PAV-104 significantly reduced structural protein production in the pellet collected from cell supernatants in a dose-dependent manner (*p* < *0.01*), but did not inhibit structural protein synthesis and steady-state levels of actin in the cell lysates (Fig. 6A, B, C). Consistent with western blot data (Fig. 6A), our NTA results showed that cells transfected with the four SARS-CoV-2 structural proteins displayed increased nanoparticle production as compared to cells transfected with empty vectors (*p* < *0.001*) (Fig. 6D), reflecting production and release of SARS-CoV-2 VLPs. PAV-104 treatment inhibited the concentration of nanoparticles in the supernatants of cells transfected with the four SARS-CoV-2 structural proteins in a dose-dependent manner (*p* < *0.01*) (Fig. 6D), while no effect on nanoparticle secretion in empty vector-transfected cell supernatants was observed (suggesting that extracellular vesicle secretion is not affected by PAV-104). These data indicate that PAV-104 specifically inhibits virus-like particle production in our model, and blocks SARS-CoV-2 replication through targeting the viral assembly/budding process.

### PAV-104 inhibits the oligomerization of the SARS-CoV-2 N

**Figure 7:**
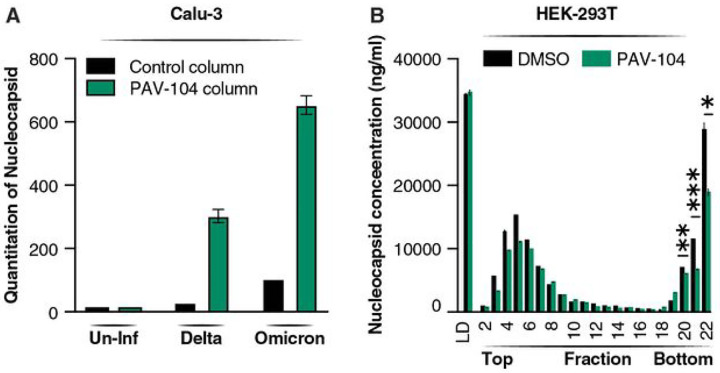
PAV-104 interacts with N and interferes with N oligomerization. (A) Quantitation of resin-bound N band density detected by western blot. DRAC experiments were performed on the PAV-104 resin column in triplicate and control resin column in singlicate from cell extracts prepared from Calu-3 cells that were uninfected (Un-Inf) or infected with SARS-CoV-2 Delta variant (Delta) or SARS-CoV-2 Omicron variant (Omicron). Material bound to the PAV-104 resin was run on gels and western blot for SARS-CoV-2 N. (B) Concentration of SARS-CoV-2 N in each fraction. Cell extracts from N-transfected cells in the presence or absence of PAV-104 were sedimented in a 10–40% glycerol gradient at 135000 g for 20 hours. Twenty-two fractions were collected and protein content analyzed using a commercial SARS-CoV-2 N protein sandwich ELISA kit (duplicate). LD=cell extracts without sedimentation. Data are representative of the results as mean ± SEM. Statistical significance was analyzed by *t* test. *p*≤*0.05* [*], *p*≤ *0.01* [**], *p*≤*0.001* [***], *p*≤*0.0001*[****].

To investigate the main drug target of PAV-104 involved in the SARS-CoV-2 viral particle assembly/budding process, drug resin affi nity chromatography (DRAC) was performed as described previously^23^. PAV-104 was coupled to the 4% crosslinked agarose resin as previously described^23^. Cellular extracts from Calu-3 cells with or without SARS-CoV-2 infection were incubated on the PAV-104 drug resin columns with or without PAV-104 covalently attached, allowing the target to bind. After washing, specifically bound material was eluted with free drug (PAV-104), followed by stripping of remaining bound material from the drug and control columns with 1% SDS. Based on western blotting with an anti-SARS-CoV-2 N antibody, negligible SARS-CoV-2 N was bound to or eluted from control columns to which infected lysates were applied, while abundant SARS-CoV-2 N was bound to and eluted from columns of PAV-104 attached to resin (Fig. 7A), indicating that SARS-CoV-2 N is a major component of the target multi-protein complex. No N reactivity was observed in columns loaded with uninfected lysates as expected. The oligomerization of the N of SARS-CoV-2 has been demonstrated to be responsible for helping virus envelope formation and particle assembly^31–33^. To determine if PAV-104 affects the oligomerization of SARS-CoV-2 N to inhibit SARS-CoV-2 viral particle assembly, cells were transfected with N in the presence or absence of PAV-104, followed by analysis with glycerol gradient ultracentrifugation and a commercial ELISA kit to determine N concentrations in fractions. N-expressing cells treated with PAV-104 showed that there is significant reduction of the N intermediate complex (*p* < *0.01*) (Fraction 20–22) as compared to DMSO treatment (Fig. 7B), indicating that the oligomerization of SARS-CoV-2 N is inhibited by PAV-104 treatment. These data support a model in which PAV-104 directly or indirectly affects the oligomerization of SARS-CoV-2 N to inhibit viral particle formation/assembly.

### PAV-104 treatment inhibits SARS-CoV-2 RNA expression and reduces ‘maturation of nucleoprotein’

**Figure 8:**
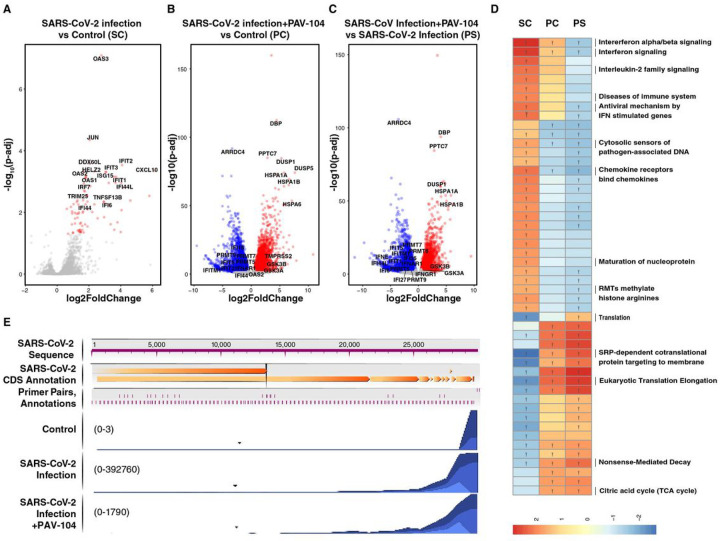
Impact of SARS-CoV-2 infection and PAV-104 treatment on the transcriptome of primary AECs. (A-C) Volcano plots showing the proportion of differentially-expressed genes (DEGs) in the setting of SARS-CoV-2 infection (SARS-CoV-2 infection *vs* Control (SC)) (A), SARS-CoV-2 infection in the presence of PAV-104 (SARS-CoV-2 infection+PAV104 *vs* Control (PC)), and SARS-CoV-2 infection in the presence of PAV-104 *vs* SARS-CoV-2 infection (PS). DEGs (FDR<0.05) with log2(fold change) > 0.5 are indicated in red. DEGs (FDR<0.05) with log2(fold change) < −0.5 are indicated in blue. The absolute value of Log2(fold change) < 0.5 and non-significant DEGs are indicated in gray. (D) Top enriched REACTOME pathways in response to SARS-CoV-2 infection or PAV-104 treatment identified using gene set enrichment analysis (GSEA). The orange and blue-colored bars in the bar chart indicate predicted pathway activation or predicted inhibition, respectively, based on enrichment-score. Y represents FDR < 0.25. (E) Sample coverage tracks from the QIAGEN genome browser depicting SARS-CoV-2 assembly. Mapped read counts of Control, SARS-CoV-2 infection, and SARS-CoV-2 infection in the presence of PAV-104 (SARS-CoV-2 infection+PAV-104) are 0 to 3, 0 to 392760, and 0 to 1790, respectively.

Finally, to understand the transcriptional impact of PAV-104 in the setting of SARS-CoV-2 infection and immunopathology, we performed RNA-seq analysis on ALI-cultured primary AECs from 5 different healthy donors infected for 36 hours with SARS-CoV-2 in the presence or absence of PAV-104. Uninfected, untreated cells (Control) were characterized as a negative control and reference for two experimental conditions: SARS-CoV-2 infection and SARS-CoV-2 infection with PAV-104 treatment. SARS-CoV-2 infection was also used as a reference for SARS-CoV-2 infection with PAV-104 treatment. Differentiallyexpressed gene (DEG) analysis showed 81 genes were significantly up-regulated by SARS-CoV-2 infection alone (Fig. 8A, Table S1), with most of them being interferon-related genes, such as OAS3, HELZ2, IFIT3, OAS1, and ISG15. SARS-CoV-2 infection in the presence of PAV-104 exhibited a dramatic impact on the host transcriptome when compared to uninfected control, with 10,255 DEGs identified (Fig. 8B, Table S2), including 5,843 down-regulated and 4,412 up-regulated genes. In addition, when compared with SARS-CoV-2 infection alone, SARS-CoV-2 infection in the presence of PAV-104 exhibited a distinct transcriptomic signature, with 9,319 DEGs identified (Fig. 8C, Table S3). GSEA pathway enrichment analysis for Reactome datasets revealed that the interferon (IFN) signaling pathway was the most up-regulated pathway by SARS-CoV-2 infection (Fig. 8D, Table S4). Virus-induced IFN signaling pathways were reversed by PAV-104 treatment. Although IFN signaling pathway play an important role in protecting the host from the spreading of SARS-CoV-2^34^, the IFN signaling pathway has also been found to be critical in initiating deleterious lung inflammatory responses^35,36^.

Of particular interest in our transcriptomic data is the set of genes related to the ‘maturation of nucleoprotein’ pathway that are selectively upregulated by SARS-CoV-2 infection but not by SARS-CoV-2 infection with PAV-104 treatment (Fig. 8D). SARS-CoV-2 nucleoprotein is found in the host cell cytosol, the nucleus and plasma membrane^37^. The ‘maturation of nucleoprotein’ signaling pathway, including oligomerization, ADP-ribosylation, phosphorylation, sumoylation, methylation and other post-translational modifications of nucleoprotein, are responsible for N movement, interaction with genomic RNAs, interaction with other proteins, and viral particle assembly^16, 38–40^. Therefore, our observation that PAV-104 suppresses the ‘maturation of nucleoprotein’ signaling pathway reinforces inhibition of viral assembly/budding as the proposed mechanism of action underlying PAV-104 anti-SARS-CoV-2 activity.

We also examined the impact of PAV-104 treatment on SARS-CoV-2 expression by aligning sequencing reads against the SARS-CoV-2 reference genome. The number of reads mapping to each region of the viral genome was calculated and interpreted to infer viral expression patterns (Fig. 8E)^28^. Consistent with the antiviral effect of PAV-104, the transcription of SARS-CoV-2 was profoundly suppressed in the presence of PAV-104. These results confirmed the highly potent antiviral activity of PAV-104 against SARS-CoV-2.

## Discussion

The rapid emergence and spreading of the SARS-CoV-2 Omicron variant that evades many monoclonal antibody therapies illustrates the need for anti-viral treatments with low susceptibility to evolutionary escape. Capsid assembly is an essential step in the viral life cycle mediated by the interaction of viral capsid proteins. Inhibition of this process can be used as a therapeutic approach; any proteins, any modifications, or any interactions which participate in or stabilize viral particle assembly in the producer cell can be manipulated to inhibit assembly, prevent release, and protect as-yet-uninfected target cells from subsequent infection.

In our prior work, we identified three small molecules, PAV-431, PAV-471, and PAV-104, as inhibitors of influenza virus assembly using our cell-free protein synthesis and viral assembly screening system^23^. We further demonstrated that PAV-104 in particular exerted highly potent antiviral effects against Nipah virus, respiratory syncytial virus, adenovirus, and human rhinovirus with minimal toxicity^23^. Here, building on these observations, we investigated the capacity of PAV-104 to inhibit SARS-CoV-2 infection. Our results show that PAV-104 inhibits SARS-CoV-2 replication in airway epithelial cells, exhibiting potent antiviral effects against a broad spectrum of circulating viral variants.

We have established that PAV-104 interferes with a post-entry step of the SARS-CoV-2 life cycle and blocks SARS-CoV-2 viral particle assembly/budding based on the following observations: 1) the chemotype of PAV-104 investigated here has no effects on early viral life cycle events (e.g. viral entry), 2) PAV-104 reduces virus release into the cell culture supernatant, 3) PAV-104 treatment does not reduce steady-state levels of cellular proteins and does not impede the translation of viral structural proteins, and 4) PAV-104 interacts with SARS-CoV-2 N and interferes with its oligomerization. Dimerization and oligomerization of SARS-CoV-2 N proteins is essential to enable associations with viral genomic RNA and other viral structural proteins (M, E, and S), playing a critical role in virus particle assembly^33,41^. In addition to viral particle assembly, the coronavirus N is required for viral mRNA and genome synthesis, viral core formation, and virus budding/envelope formation^42^. Based on our data revealing interaction between PAV-104 and SARS-CoV-2 N, PAV-104 treatment may also affect other key post-entry steps in the viral cycle beyond virus assembly.

Previously, we showed that PAV-104 bound a small subset of the known allosteric modulator 14-3-3, itself implicated in the interactome of SARS-CoV-2^23,43,44^. Binding of phosphorylated SARS-CoV N to the host 14-3-3 protein in the cytoplasm was reported to regulate nucleocytoplasmic N shutting and other functions of N^45^. In addition, human 14-3-3 proteins were reported to bind the mutational hotspot region of SARS-CoV-2 N and modulate SARS-CoV-2 N phosphoregulation^46^. In accordance with these observations, our transcriptomic data showed that PAV-104 treatment negatively regulates the ‘maturation of nucleoprotein’ signaling pathway of SARS-CoV-1/2. For example, sumoylation of SARS-CoV-2 N protein can enhance its interaction affi nity with itself and is critical for its nuclear translocation, which is in turn critical for N-mediated viral RNA genome packaging and interaction with M protein^40,47^. Phosphorylation of SARS-CoV-2 N protein was reported to be responsible for its localization, phase-phase separation and interaction with host factors^31^. The precise manner in which PAV-104 affects the post-translational modification of SARS-CoV-2 N warrants additional investigation, which may reveal novel antiviral mechanisms and pharmacological targets.

Our transcriptomic analysis also revealed that PAV-104 treatment of infected cells reduced the expression of specific IFN-regulated genes and reversed SARS-CoV-2 induction of the IFN signaling pathway. IFN signaling is critical to antiviral responses^34,48^. To counteract host defense, multiple studies have demonstrated that SARS-CoV-2 uses a multitude of mechanisms to avoid type-I IFN-mediated immune responses^49^. On the other hand, robust type I IFN responses have been associated with severe COVID-19 disease, and may exacerbate hyperinflammation during the development of severe COVID-19^50,51^. Therefore, beyond inhibition of viral replication, PAV-104 may exert adjunctive anti-inflammatory effects via selective suppression of interferon pathway members that enhances its clinical potential as a therapeutic for COVID-19.

In summary, our findings demonstrate that PAV-104, a host-targeted pan-viral small molecule inhibitor, is a promising therapeutic candidate for SARS-CoV-2.

## Methods

### Cell lines

Human lung adenocarcinoma epithelial Calu-3 cells (ATCC-HTB-55) were cultured in Eagle’s Minimum Essential Medium (EMEM). African green monkey kidney Vero E6 cells (ATCC-CRL-1586) and human kidney HEK29T cells (ATCC-CRL-3216) were cultured in Dulbecco’s Modified Eagle’s medium (DMEM). All media were supplemented with 10% FBS and 1% penicillin/streptomycin. Vero E6 cells stably expressing TMPRSS2 (Vero E6-TMPRSS2) were established and cultured in DMEM in the presence of puromycin (1μg/ml).

All cells had been previously tested for mycoplasma contamination and incubated at 37°C in a humidified atmosphere with 5% CO2.

### Primary airway epithelial cells (AECs)

Primary AECs were obtained and cultured as previously described^28^. Briefly, Human unused donor tracheobronchial tissue was obtained at the time of lung transplant. The tissue was washed and placed in DMEM with 0.1% protease and antibiotics overnight at 4°C. The next day, the solution was agitated, and the remaining tissue was removed. Cell pellets were treated with 0.05% trypsin-EDTA, then filtered through a cell strainer. Cells were plated onto 6mm/0.4mm Transwell ALI insert after treatment with FNC coating mixture. 10% FBS in DMEM and ALI media were added in equal volumes to each basal compartment and cultures were incubated at 37°C with 5% CO_2_. The next day, the media was removed and both compartments were washed with PBS and antibiotics. ALI media was then added to each basal compartment and changed every three days for at least 28 days until differentiated airways were ready for use.

### Viruses

The severe acute respiratory syndrome coronavirus 2 (SARS-CoV-2) strains USA-WA1/2022, lineage P.1, lineage B.1.617.2, and lineage B.1.1.529 were obtained from BEI Resources of the National Institute of Allergy and Infectious Diseases (NIAID) and propagated in Vero E6-TMPRSS2 cells. Virus titer was measured in Vero E6 cells by TCID_50_ assay. All the studies involving live viruses were conducted in the Vitalant Research Institute BSL-3 under approved safety protocols.

### Ethics Statement

The studies involving human participants were reviewed and approved by the Human Research Protection Program, University of California, San Francisco. The patients/participants provided their written informed consent to participate in this study.

### Preparation of PAV-104

The synthetic method of PAV-104 was illustrated in Fig. 1. To a solution of aldehyde1 (10 g, 65.79 mmol, 1.0 eq) in toluene was added 2,4-dimethoxybenzyl amine 2 (10.99 g, 65.79 mmol, 1.0 eq) and the reaction mixture was heated at 80°C for 24 h. Solvent was removed and the residue was taken in MeOH and cooled using an ice bath. Then sodium borohydride (4.97g, 131.58 mmol, 2.0 eq) was added slowly and the reaction mixture was stirred at room temperature for 12 h. Solvent was removed and residue was taken in ethyl acetate and then sat. NaHCO3 was added and stirred for 1 h. Organic layer was separated, dried (MgSO4) and solvent was removed to give amine 3, which was used in the next step without further purification.

To a solution of the crude amine 3 (5.0 g, 19.1 mmol, 1.0 eq) in DMF (25 mL) were added acid 4 (3.17 g, 19.1 mmol, 1.0 eq), HATU (8.7 g, 22.92 mmol, 1.2 eq,), and DIEA (12.32 g, 95.5 mmol, 5.0 eq) and the reaction mixture was stirred at room temperature for 12 h. The reaction mixture was then diluted with ethyl acetate (EtOAc) and washed with 10% aqueous HCl (1X), sat. NaHCO3 (1X) and water (3X). Organic layer was collected, dried (MgSO4) and evaporated to give a residue, which was taken in MeOH and then K2CO3 (2.64 g. 19.1 mmol, 1.0 eq) was added and stirred at room temperature for 12 h. Solvent was removed and the residue was taken in Ethyl acetate and washed with 10% HCl (1X). Organic layer was separated, dried and solvent was removed to give a residue, which was purified by column chromatography (EtOAc/Hexane) to give compound 5.

To a stirred solution of compound 5 (1.0 g, 2.22 mmol, 1.0 eq) and cesium carbonate (1.08 g, 3.33 mmol, 1.5 eq) in DMF (15 mL) was added methyl 4-(chloromethyl) benzoate 6 (450 mg, 2.44 mmol, 1.2 eq) and the reaction mixture was stirred at room temperature for 18 h. The reaction mixture was diluted with ethyl acetate and washed with water (3x). Organic layer was dried and concentrated to give crude product 7. The crude compound 7 was stirred in a 1:1 mixture of TFA: DCM for 12 h. Concentration followed by chromatography purification (Hexane/EtOAc) provided compound 8.

To a stirred solution of compound 8 (0.84 mmol, 1.0 eq) in 3:1 mixture of THF: H_2_O (12 mL) was added LiOH (40 mg, 1.68 mmol, 2.0 eq) and the reaction mixture was stirred at 65°C for 12 h. The reaction mixture was evaporated under vacuum to give a residue, which was stirred in a mixture of 10% aqueous HCl and ethyl acetate for 30 min. Organic layer was collected, washed (H_2_O, 1X), dried and concentrated to give crude acid 9.

To a solution of the amine 10 (68 mg, 0.552 mmol, 1.2 eq) in DMF (25 mL) were added acid 9 (200 mg, 0.46 mmol, 1.0 eq), HATU (210 mg, 0.552 mmol, 1.2 eq,) and DIEA (0.300 mg, 2.3 mmol, 5.0 eq). The reaction mixture was stirred at room temperature for 12 h. The reaction mixture was then diluted with EtOAc and washed with 10% aqueous HCl (1X), sat. NaHCO3 (1X) and water (3X). Organic layer was collected, dried (MgSO4) and evaporated to give a residue, which was purified by column chromatography (EtOAc/Hexane) to give PAV-104.

### Drug cytotoxicity assay

The cytotoxic effect of PAV-104 on Calu-3 cells was measured using an MTT assay kit (Abcam, ab211091) following the manufacturer’s instructions. In brief, Calu-3 cells were seeded in 96-well cell culture plates. Appropriate concentrations of PAV-104 were added to the medium (0–5000 nM). After 48 hours, the media was removed and 100 μl MTT reagent (1:1 dilution in DMEM medium (serum free)) was added to each well and incubated for 3 h at 37°C. Then the medium was removed, and 150 μl MTT solvent was added into each well. Quantification was performed by reading absorbance at OD = 590 nm. The data from three independent experiments was used to calculate the CC_50_ by nonlinear regression using GraphPad Prism 8.0 software.

### SARS-CoV-2 infection and drug administration

Calu-3 cells were seeded at 0.5 × 10^6^ cells per well in 0.5 ml volumes using a 24-well plate, or were seeded at 1 × 10^5^ cells per well in 0.1 ml volumes using a 96-well plate. The following day, cells were pretreated with or without PAV-104 or remdesivir for one hour. Then viral inoculum (MOI of 0.01; 500 μl/well or 100μl/well) was prepared using EMEM containing indicated concentrations of PAV-104 or remdesivir and added to the wells. The inoculated plates were incubated at 37°C with 5% CO_2_. At indicated infection time points, supernatants were collected and stored at −80°C. Cells were lysed with TRizol (Thermo Fisher Scientific, 15596026) for RNA extraction.

For infection of primary AECs in ALI culture, cells were pretreated with PAV-104 in the basal compartment for one hour. SARS-CoV-2 (diluted in ALI-culture medium, MOI = 0.1) was added to the apical chamber of inserts (250 μl) and the basal compartment (500 μl). Then the cultures were incubated for 2 hours at 37°C (5% CO_2_) to enable virus entry. Subsequently, the cells were washed and fresh ALI medium (500 μl) containing PAV-104 was added into the basal compartment. Cells were incubated at 37°C (5% CO_2_) and harvested for analysis at 36 hours post-infection.

### Viral titer by TCID assay

Virus production in the supernatant was measured by quantifying TCID_50_. Vero E6 cells were plated in 96-well plates at 5 × 10^4^ cells per well. The next day, supernatants collected from Calu-3 cells were subjected to 10-fold serial dilutions (10^1^ to 10^11^) and inoculated onto Vero E6 cells. The cells were incubated at 37°C with 5% CO_2_. Three to five days post infection, each inoculated well was evaluated for presence or absence of viral CPE. TCID_50_ was calculated based on the method of Reed and Muench.

### RT-qPCR

Total RNA was extracted using TRIzol reagent according to the manufacturer’s instructions. Reverse transcription was performed using RevertAid First Strand cDNA Synthesis Kit (Thermo Fisher Scientific, K1622) in accordance with the manufacturer’s instructions. RT-qPCR was performed for each sample using Taqman Universal Master mix II, with UNG (Thermo Fisher Scientific, 4440038) on a ViiA7 Real time PCR system. Primers and probes for detection of the *RNaseP* gene and SARS-CoV-2 *nucleocapsid* (*N*) gene were obtained from IDT (2019-nCoV RUO Kit (Integrated DNA Technologies, 10006713)). The expression level of the *N* gene was determined relative to the endogenous control of the cellular *RNaseP* gene.

### RNA-sequencing analysis

RNA concentration and quality was measured using High Sensitivity RNA ScreenTape Analysis (Agilent, 5067 − 1500). cDNA libraries were constructed and sequencing was performed by Novogene using their mRNA sequencing protocol. The raw RNA sequencing data were aligned to the human genome (GRCh38) using STAR (version 2.7.3a). Analysis of differential expression was performed using DESeq2 according to a standard protocol. Genes with adjusted *P*-value < 0.05 were considered as significantly differentially expressed. Gene set enrichment analysis was performed using the fgsea package (version 1.22.0) in R. The Reactome database (version 7.5.1) was downloaded from MSigDB (https://www.gsea-msigdb.org).

### Immunofluorescence microscopy and image analysis

Cells were fixed and permeabilized with cold methanol : acetone (1:1) for 10 min at 4°C according to our previous method^28^. In brief, cells were then incubated with blocking buffer (5% goat serum (Seracare Life Sciences Inc, 55600007)), a primary antibody (monoclonal rabbit anti-SARS-CoV-2 N antibody (GeneTex, GTX135357)), a secondary antibody (Goat anti-Rabbit IgG (H + L) secondary antibody, FITC (Thermo Fisher, 65–6111)), and DAPI (Thermo Fisher Scientific, D1306). Images were acquired using a fluorescence microscope.

To measure the frequency of infected cells, randomly-selected areas were imaged. Each treatment had three replicates. The FITC-positive cells and DAPI-positive cells were quantified using CellProfiler software as previously described. The same threshold value was applied to the images of each area.

Quantification of the western blots was carried out with Image J software.

### Production of SARS-CoV-2 virus-like particles (VLPs)

HEK293T cells were seeded in T75 cell culture flasks. The next day, cells were transfected with empty pcDNA3.1 plasmid or pcDNA3.1 plasmid encoding the SARS-CoV-2 M (Addgene-158078), E (Addgene-158080), N (Addgene-158079), and S proteins (Addgene-158074), as indicated. 1 μg of each plasmid was used, with 5 μg of total plasmid in each transfection, normalized using empty vectors, in 400 μl Opti-MEM and 18 μl of PEI. The transfection mixture was incubated at room temperature for 15 min and dropped into the HEK293T cells. Six hours post transfection, the media was removed and supplemented with fresh medium containing PAV-104 at indicated concentrations. The supernatant and cell lysate were collected after 60 hours. For the purification of SARS-CoV-2 VLPs, the supernatant was passed through a 0.45 μm syringe filter, then loaded on top of a 20% sucrose cushion in PBS, and ultracentrifuged at 30,000 rpm in an SW41 rotor for two hours. VLP-containing pellets were washed with ice cold PBS and resuspended in SDS loading buffer, followed by sonication in an ice-water bath. Or VLP-containing pellets were resuspended in PBS (passed through 0.22 μm syringe filter) for quantification by NTA. Cells were lysed in RIPA buffer (Thermo Fisher Scientific, 89900) and sonicated in an ice-water bath.

### Immunoblots of SARS-CoV-2 VLPs

Total protein in pellet and cell lysate samples were separated by SDS-PAGE, and subsequently electrotransferred onto a supported PVDF membrane. Membranes were cut and probed for M with rabbit anti-SARS-CoV-2 M (Thermo Fisher Scientific, PA1–41160), N with rabbit anti-SARS-CoV-2 N (Rockland Immunochemicals, 200–401-A50), E with rabbit anti-SARS-CoV-2 E (Thermo Fisher Scientific, PA5–112047), and S with rabbit anti-SARS-CoV-2 S1/S2 (Thermo Fisher Scientific, PA5–112048) or with mouse anti-SARS-CoV-2 spike (Genetex Inc, GTX632604). Goat anti-rabbit IgG HRP and goat anti-mouse IgG HRP secondary antibodies were used as appropriate. β-actin was used as a cell lysate and pellet loading control by probing membranes with rabbit anti-human β-actin, conjugated with HRP (Cell Signaling Technology, 12620). All antibodies were diluted in 5% milk and membranes were washed with Tween 20 washing buffer (Thermo Fisher Scientific, J60304.K3). Chemiluminescent signal was visualized using SuperSignal West Femo Substrate (Thermo Fisher Scientific, PI34094) or using ECL Blotting Reagents (SIGMA, GERPN2109), and imaged using ImageQuant LAS 4000.

### Quantification of VLPs by nanoparticle tracking analysis (NTA)

VLP-containing pellets were diluted in PBS (passed through 0.22 μm syringe filter) to a concentration in the range of 10^7^–10^9^/ml and examined using a NanoSight NS300 (NanoSight Ltd) equipped with a 405 nm laser. Five 60 s-long videos were taken for each sample with camera level 16 and the detection threshold set at 5. Raw data of particle movement and laser scattering were analyzed using NTA software (version 3.3, NanoSight Ltd). The output data were presented as nanoparticle concentration and size.

### Drug Resin Affinity Chromatography (DRAC)

Drug Resin Affi nity Chromatography experiments were performed where 30 μl of extract prepared from Calu3 cells under different infection and treatment conditions were adjusted to a protein concentration of approximately 2.3 mg/ml in column buffer and supplemented with an “energy cocktail” (to a final concentration of 1mM rATP, 1mM rGTP, 1mM rCTP, 1mM UTP, 4mM creatine phosphate, pH 7.6) and 5 μg/mL creatine kinase) and incubated on a column containing 30 μl of affi-gel resin coupled to either PAV-104 or a 4% agarose matrix (control) for one hour at room temperature. The PAV-104 resin conditions were run side-by-side in triplicate, while the control resin conditions were done in single point. The flowthrough material was collected, and the resin was washed with 1.5 mL column buffer then eluted with 100 μl PAV-104 plus the energy cocktail at room temperature for two hours then stripped with 100 μl 1% SDS. The eluate and SDS-stripped material run on agarose gels and are analyzed by western blot for SARS-CoV-2 N protein (Rockland Immunochemicals, 200–401-A50).

### Glycerol gradient sedimentation and ELISA-based assessment of SARS-CoV-2 N

Cell extracts from SARS-CoV-2 N-transfected HEK-293T cells in the presence or absence of PAV-104 were centrifuged at 15000 g for 10 mins at 4°C to obtain the supernatant. 200 μl of supernatant were loaded on the top of a 5 ml continuous 10–40% glycerol gradient in lysis buffer (v/v, Pierce IP Lysis Buffer (Thermo Fisher, 87787)) prepared using the Gradient Master machine (Biocomp, Gradient Station). After concentration at 135000 g for 20 h at 4°C in a SW55 rotor (Beckman Coulter), 22 fractions of 250 μl were collected from the top to the bottom of the gradient.

Proteins were assessed by the commercial SARS-CoV-2 N protein sandwich ELISA kit (GeneTex, GTX535824) following manufacturer’s instructions. In brief, each fraction was diluted to 1:1000 using assay dilute reagent. 50 μl of each standard and samples were added into the appropriate wells, then incubated at room temperature for 2 hours. The solutions in the wells were aspirated and wells were washed with a washing buffer six times. Then the conjugate solution was added and incubated at room temperature for 1 hour. The solutions in the wells were aspirated and wells were washed with a washing buffer six times once again. TMB solution was added to the wells and incubated in darkness for 15 mins at room temperature. Stop solution was added to each well. Finally, optical density at 450 nm was read within 15 mins.

### Statistical analysis

Statistical analysis was performed using GraphPad Prism version 8 software. Data were presented as means ± SEM or median. Data were analyzed for statistical significance using an unpaired or paired Student’s *t* test to compare two groups, or using a paired *t* test. Only *p* values of 0.05 or lower were considered statistically significant (*p* > *0.05* [ns], *p* ≤ *0.05* [*], *p* ≤ *0.01* [**], *p* ≤ *0.001* [***], *p* ≤ *0.0001* [****]).
